# Road Salts as Environmental Constraints in Urban Pond Food Webs

**DOI:** 10.1371/journal.pone.0090168

**Published:** 2014-02-26

**Authors:** Robin J. Van Meter, Christopher M. Swan

**Affiliations:** 1 Marine Estuarine Environmental Sciences Program, University of Maryland, Baltimore, Maryland, United States of America; 2 Department of Geography and Environmental Systems, University of Maryland, Baltimore, Maryland, United States of America; Northwest Fisheries Science Center, NOAA Fisheries, United States of America

## Abstract

Freshwater salinization is an emerging environmental filter in urban aquatic ecosystems that receive chloride road salt runoff from vast expanses of impervious surface cover. Our study was designed to evaluate the effects of chloride contamination on urban stormwater pond food webs through changes in zooplankton community composition as well as density and biomass of primary producers and consumers. From May – July 2009, we employed a 2×2×2 full-factorial design to manipulate chloride concentration (low = 177 mg L^−1^ Cl^−/^high = 1067 mg L^−1^ Cl^−^), gray treefrog (*Hyla versicolor*) tadpoles (presence/absence) and source of stormwater pond algae and zooplankton inoculum (low conductance/high conductance urban ponds) in 40, 600-L mesocosms. Road salt did serve as a constraint on zooplankton community structure, driving community divergence between the low and high chloride treatments. Phytoplankton biomass (chlorophyll [a] µg L^−1^) in the mesocosms was significantly greater for the high conductance inoculum (*P*<0.001) and in the high chloride treatment (*P* = 0.046), whereas periphyton biomass was significantly lower in the high chloride treatment (*P* = 0.049). Gray treefrog tadpole time to metamorphosis did not vary significantly between treatments. However, mass at metamorphosis was greater among tadpoles that experienced a faster than average time to metamorphosis and exposure to high chloride concentrations (*P* = 0.039). Our results indicate differential susceptibility to chloride salts among algal resources and zooplankton taxa, and further suggest that road salts can act as a significant environmental constraint on urban stormwater pond communities.

## Introduction

Regional and local factors such as dispersal, competition, timing of colonization events and environmental filters in concert determine community composition [1]. In particular, environmental constraints can present very stressful conditions, such as high pollutant loads or changes in hydroperiod, that exceed species’ tolerances and prevent successful colonization to new habitats [1]–[2]. Environmental constraints or stressors, as detailed and studied herein, refer to anthropogenic influences to aquatic ecosystems. In systems that experience fluctuating environmental stress, such as pulses of contaminant loads, food web dynamics can be very cyclic when species are well adapted to specific abiotic conditions. Species adapted to opposite ends of the contaminant spectrum will be intermittently dominant within the community as the environment changes or they may even be lost from the system completely [1], [3]. However, species that are able to withstand intense environmental stresses may do so at the expense of being less competitive when biotic factors regulate community composition [4]. Pollutants of rising concern can serve as environmental filters by limiting community composition to those species that are able to withstand the stress. In human dominated landscapes, environmental constraints may be the prevailing local factor in determining community assembly.

The importance of environmental constraints in structuring aquatic communities has been emphasized within the last decade as concerns of habitat alteration and degradation continually increase. Studies of freshwater zooplankton communities show that variability in water chemistry is often the best predictor of community composition. Gradients or changes in pond turbidity, hydroperiod, salinity or conductivity and pH can result in distinct zooplankton assemblages [5]–[9]. Similar water quality parameters are reported as main factors in community assembly among diatoms [10], phytoplankton [11], aquatic invertebrates [6] and amphibians [4]. To better predict community composition in rapidly changing landscapes, gaining a better understanding of the relationship between species tolerance thresholds and relevant environmental constraints is essential.

Across the northeast United States, freshwater salinization is becoming a year-round phenomenon due to long-term storage and transport of dissociated road salt ions (e.g., chloride, sodium, magnesium) in groundwater and soils [12]–[15]. This emerging environmental contaminant is of particular concern in urban areas where natural wetland acreage continues to decline and man-made stormwater ponds are common watershed features [16]. While stormwater detention ponds are designed to accumulate a myriad of contaminants, recent research has highlighted the susceptibility of these urban aquatic ecosystems to road salt deicers resulting in freshwater salinization [17]. An unintended consequence of installing numerous stormwater detention ponds is that many organisms have been documented making use of these man-made habitats despite their extremely poor water quality [18–24 and others]. Given their position within urbanized landscapes, stormwater detention ponds present a unique system for exploring road salts as an environmental constraint on aquatic community structure.

Road salt exposure at sub-lethal concentrations can result in significant direct and indirect effects within a community, as well as alter ecosystem structure of freshwater habitats. Van Meter et al. [25] obtained algae, zooplankton and frogs from pristine wetland communities and reported a decline in zooplankton densities in experimental pond mesocosms at chloride concentrations known to be sub-lethal to gray treefrog larvae. As a result of the loss of this algal grazer from the ecosystem, gray treefrog larvae gained access to algal resources and metamorphosed faster and at a larger mass [25]. Our current study expands on this previous work by considering the potential for adaptation and altered community structure among urban stormwater pond communities that experience high chloride levels year-round. Recent work by Petranka and Doyle [26] indicated that road salts can alter seasonal pond communities in favor of salt tolerant insects such as mosquitoes, which may serve as competitors to larval amphibians and zooplankton. Jose de Paggi et al. [27] described unique zooplankton communities between the inlet and outlet of an urban stormwater pond receiving chloride inputs, and Olding [28] found distinct algal assemblages between stormwater ponds retaining different chloride concentrations. Given that road salt contamination is a rising concern to freshwater ecosystems and differential susceptibility exists among aquatic organisms to these contaminants, exploring patterns in stormwater pond communities is essential in understanding the ecology of the urban landscape.

While several studies have explored the effects of chloride contamination on pond populations and communities, none have evaluated such effects in habitats experiencing perpetual disturbance through contaminated runoff. The purpose of this study was to evaluate the impact of a hypothesized environmental constraint, road salt (low and high chloride treatments), on the community structure in experimental stormwater pond systems assembled from different colonist pools (i.e., zooplankton and algal inoculum source) and in the presence of a major biotic factor, gray treefrog (*Hyla versicolor*) tadpoles. While previously published studies have explored experimental road salt effects on pristine pond communities [25]–[26] and have surveyed community structure in stormwater ponds with high chloride levels [27]–[29] as detailed above, our experiment was designed to evaluate whether chloride salts serve as an environmental stressor to pond communities thriving in urban ecosystems where chloride levels are consistently elevated for much of the year. Our hypothesis was that road salt inputs do serve as an important environmental constraint on pond communities and that urban pond communities experiencing continual salt stress would contain a species pool that is more tolerant of elevated chloride.

## Materials and Methods

### Ethics Statement

This research was approved by the University of Maryland Baltimore County Institutional Animal Care and Use Committee (protocol #CS070210710) and carried out in accordance with the Association of Ichthyologists and Herpetologists Guidelines for Use of Live Amphibians and Reptiles in Field and Laboratory Research. Permission to collect gray treefrogs for this study was given by the Maryland Department of Natural Resources (Scientific Collecting Permit #46158). Collection of periphyton, phytoplankton and zooplankton did not require specific permissions as these samples were obtained from public stormwater ponds. This study did not involve the use or collection of endangered or protected species.

Our experimental design was a 2×2×2 full factorial (Table 1) with a chloride treatment as an environmental effect (high, low), tadpole treatment as a biotic effect (present, absent), and algae/zooplankton inoculum treatment as the colonist pool (low conductance pond, high conductance pond) applied randomly to 40 polyethylene mesocosms, each 600 L in volume. These mesocosms were arranged in a 5×8 grid in a grassy, open field at the University of Maryland, Baltimore County (UMBC), Baltimore, Maryland, USA. The open field provided uniformity in the amount of ambient light, wind and rain that the mesocosms received over the course of the study. On May 6–7, 2009, we filled the mesocosms with tap water and allowed the water to age for 24–48 hours before adding 12.5 g of rabbit chow to supply nutrients, 95 g of dry leaves (*Quercus* sp.) to provide cover, and 10 unglazed ceramic tiles, each 103.2 cm^2^, as a surface for periphyton colonization in all mesocosms. We obtained inoculum for periphyton, phytoplankton and zooplankton from two stormwater detention ponds in the Red Run Watershed, Owings Mills, Baltimore County, Maryland. These ponds were part of two stormwater detention pond surveys between 2007–2009 (ponds E94 and H85; [29]–[30]. Gallagher et al. [30] analyzed sediment samples for trace metals and polycyclic aromatic hydrocarbons and water samples for major ions in 68 stormwater ponds in the Red Run Watershed, including the two ponds used in this study. A strong correlation between chloride ions and conductivity was reported for all ponds sampled [30]. Pond E94 was classified as having a low conductance (average specific conductance from April – June 2009 = 153±15.5 (SE) µS, n = 4) while pond H85 was classified as having a high conductance (average specific conductance from April – June 2009 = 1924±303 (SE) µS, n = 4; [25]. Before adding to the mesocosms, we filtered the pond water inoculum through a 500 µm sieve to remove larger invertebrates and debris. We added 5 L of low conductance inoculum to 20 mesocosms and 5 L of high conductance inoculum to the remaining 20 mesocosms on May 8, 14 and 20, 2009 for a total of 15 L to each mesocosm. We covered each mesocosm with 60% shade cloth, secured covers with drawstrings to prevent insect colonization, then allowed them to remain undisturbed until June 7, 2009.

**Table 1:** 2×2×2 factorial design applied to 40 outdoor mesocosms (n = 5 per treatment combination) with a road salt treatment (low or high), tadpole treatment (present or absent) and inoculum treatment (collected from low or high conductance stormwater pond).

Mesocosm #	Road Salt	Tadpoles	Inoculum Source
8,20,22,30,31	Low	Absent	Low
7,14,21,27,35	Low	Present	Low
2,9,16,33,40	Low	Absent	High
3,6,15,29,32	Low	Present	High
5,11,18,24,37	High	Absent	High
1,12,25,34,38	High	Present	High
10,13,17,26,39	High	Absent	Low
4,19,23,27,36	High	Present	Low

This study was designed to be sublethal to gray treefrog (*Hyla versicolor*) larvae; therefore, the higher chloride concentration used reflected the uppermost limit for successful embryonic and larval survival as determined by Brand et al. [31]. The stormwater ponds used to collect inoculum were representative of those in Red Run Watershed that can support successful amphibian development and survival [23]. We measured specific conductance (µS) and temperature (°C) for each mesocosm 3 times between June 6 and July 21 (study day 0, 24 and 45), 2009 using a YSI-85 handheld probe. Specific conductance of the aged tap water added to each mesocosm before chloride addition was 260 µS (68.5 mg L^−1^ Cl^−^) (for chloride-conductivity equation see Karraker [32]). On May 8, 2008 we oven-dried granular sodium chloride (99% NaCl) at 30°C for two hours, then added 89.7g NaCl to 20 mesocosms receiving the low chloride treatment and 823.8g NaCl to the remaining 20 mesocosms receiving the high chloride treatment. This resulted in a final chloride concentration of 177.2mg L^−1^ in 500 L of water and average specific conductance of 675.5±4.3 (SE) µS for the 20 mesocosms receiving the low chloride treatment and a final chloride concentration of 1067mg L^−1^ and average specific conductance of 3016±21.9 (SE) µS for the 20 mesocosms receiving the high chloride treatment.

On May 29, 2009 we collected 5 amplexed pairs of gray treefrogs (*Hyla versicolor*) from a stormwater detention pond in the Red Run Watershed and transported them to UMBC in pond water collected from the breeding site (specific conductance 270 µS). After oviposition was complete, we removed adult treefrogs and allowed the embryos to develop in aquaria until they reached Gosner stage 17 [33]. To mix clutches, we transferred all treefrog larvae into three large plastic bins filled with 50 L of aged tap water (specific conductance 315 µS). Treefrog larvae were reared in these bins until they reached the free feeding stage (Gosner stage 25; [33]). On June 7, 2009 (day 1), we added 30 gray treefrog (*Hyla versicolor*) tadpoles (Gosner stage 25; [33]) to the 20 mesocosms assigned the tadpole treatment.

The first gray treefrog metamorph emerged from the experimental mesocosms at day 25 (July 1, 2009) after tadpole addition to the mesocosms. After emergence of the first metamorph we performed daily checks for metamorphs until the study was terminated at day 54 (July 30, 2009). Upon emergence, all metamorphs were housed in the laboratory at UMBC until tail resorption was complete (Gosner stage 46), at which point all individuals were weighed. On the last day of the study, we collected, measured wet weight and staged [33] any remaining tadpoles in each mesocosm.

We analyzed both periphyton and phytoplankton samples for chlorophyll [a] concentration (µg L^−1^) using the non-acidified chlorophyll [a] module on a Trilogy fluorometer [34]. On days 3, 24 and 45 (June 9, June 30 and July 21, 2009), we collected periphyton samples from each mesocosm by scraping the entire surface of one ceramic tile and bringing the sample to a total volume of 100 mL using distilled water. We vacuum filtered a 25 mL subsample onto a 47 mm Whatman GF/F [35]. On days 5, 26 and 47 (June 11, July 2 and July 23, 2009), we collected phytoplankton samples by submerging a 1.27 cm diameter PVC tube spanning the entire depth of the water column in three haphazard places within each mesocosm. We then filtered 100 mL of the resulting composite water sample onto a 47 mm Whatman GF/F. We stored all filtered periphyton and phytoplankton samples in 90% acetone in the dark at 4°C for a maximum of 96 hours before analysis.

We collected zooplankton samples on day 47 by submerging a 5.1 cm diameter PVC tube vertically into the water column making sure to sample the entire depth of the mesocosm and collecting 2 L of water. We poured this water sample through a 63 µm sieve and stored zooplankton samples in 80% ethanol. To estimate zooplankton density, we standardized the volume of each mesocosm sample by bringing total volume to 500 mL using distilled water. We homogenized the 500 mL sample by stirring with a plastic pipette, and then viewed 10, 2 mL subsamples under a compound microscope using a 1 mm^2^ gridded Sedgewick Rafter. We sorted and counted the zooplankton into the following groups for density estimates: adult copepods, copepod nauplii (juveniles), ostracods, rotifers and daphnia. To estimate zooplankton species composition across treatments, we further identified zooplankton to order or family, and in some occasions to genus and species when possible. For species identification, we brought each sample to a total of 50 mL and viewed 1, 2 mL subsample to generate a species area curve for each mesocosm.

We measured dissolved oxygen (mg L^−1^) on one sampling date, day 24, using a YSI 20 handheld meter. Although we cannot rule out large changes in dissolved oxygen or pH over the course of the study, our previous research in these artificial pond systems suggested there would be no dramatic shifts in either of these parameters and no systematic shifts with our study treatments [25]. Given the high nitrogen content of waste released by developing tadpoles [36], we also collected water samples on day 45 to obtain nitrate estimates. We filtered water samples using 25 mm Whatman GF/F, prepared samples using LaMotte Nitrogen kits and analyzed for absorbance (AB) using the absorbance module on a Trilogy fluorometer [34]. To obtain final nitrate estimates in mg L^−1^, we made sodium nitrate standards and created a standard curve of sodium nitrate (g L^−1^) versus absorbance.

### Statistical Analysis

We performed all statistical analyses related to algal biomass and zooplankton density, as well as tadpole time to and mass at metamorphosis, in SAS version 9.1.3 [37]. To determine the effects of the tadpole, chloride and inoculum treatments on phytoplankton and periphyton we used a repeated measures analysis of variance (RMANOVA) with compound symmetry covariance structure to account for any correlation among experimental units. We used a 3-way analysis of variance (ANOVA) to determine the effects of tadpole, chloride and inoculum treatments on zooplankton density. All periphyton, phytoplankton and zooplankton data was log_10_ transformed to maintain normality and homogeneity of variances. When necessary, we grouped residual variances by treatments using the GROUP option in PROC MIXED following the method of Littell et al. [38]. We performed a logistic regression using PROC GENMOD to assess the impact of the chloride treatment on percent tadpole survival. We used the scale = deviance option within PROC GENMOD to handle over-dispersion of data. Mass at metamorphosis among gray treefrogs was analyzed using analysis of covariance (ANCOVA) with chloride and inoculum treatment modelled as a class variable and time to metamorphosis as a continuous variable. Time to metamorphosis was analyzed using a Generalized Linear Model (GLM) with chloride and inoculum treatment modelled as a class variable and survival as a continuous variable. When survival was found to have no significant effect, time to metamorphosis was analyzed using ANOVA.

To evaluate any effects of the tadpole, chloride and inoculum treatments as well as study day on conductivity, temperature or dissolved oxygen we performed factorial ANOVA with Type 3 Sums of Squares analyses using the aov() and drop1() functions in R statistical computing environment [39]. To determine if chloride served as an environmental constraint on zooplankton community composition, we also performed analyses in R. We approached analyzing zooplankton community composition in two ways. First, we examined compositional divergence using the adonis() function on a Bray-Curtis dissimilarity matrix. This analysis is a non-parametric permutation MANOVA that determines if dissimilarity was related to the factorial design. Second, since we hypothesized that road salt served as a significant constraint on zooplankton communities, we analyzed turnover, or variation in community structure among mesocoms within and across treatments. This was carried out using the betadisper() function in R, which is an analysis of multivariate homogeneity of group dispersions, an analogue of the univariate Levene’s test for homogeneity of variance [40]. If the presence of deicer is an environmental filter, variation in community structure, or taxonomic turnover, should be lower among mesocosms receiving road deicer. This procedure is a permutation test based on 999 randomizations, resulting in a p-value associated with an F-test comparing the two salt treatment groups. Results were visualized using non-metric multidimensional scaling based on the Bray-Curtis dissimilarity matrix. Points closer together represent communities more similar in taxonomic composition than those further apart.

## Results

### Abiotic Environmental Variables

Specific conductance (µS) in both the low and high chloride treatments remained stable over the duration of the study, with some minor dilution across the study period due to periods of heavy rainfall (Low = 568±6.7 (SE) µS; High = 2856±14.7 (SE) µS). Mean specific conductance (µS) and water temperature (°C) measured three times between days 0 and 45 are reported in Table 2 along with dissolved oxygen (mg L^−1^) as measured once on day 24 and nitrate (mg L^−1^) values measured on day 45 (Table 2; Table S1). There were only nominal concentrations of nitrate present in the mesocosms at the end of the study.

**Table 2:** Mean abiotic environmental variables measured in all mesocosms (n = 20 per chloride treatment) from study days 0–47± standard error.

	Chloride Treatment	
Environmental Variable	Low	High
Specific Conductance (µS)		
Day 0	675.5±4.3	3016±28.7
Day 24	536.5±3.1	2815±16.8
Day 45	513.8±2.9	2705±16.9
Water Temperature (°C)		
Day 0	22.2±0.1	21.9±0.1
Day 24	25.5±0.1	25.5±0.0
Day 45	26.3±0.1	25.8±0.1
Nitrates (mg/L)		
Day 45	<0.003	<0.004
Dissolved Oxygen (mg/L)		
Day 24	5.2±0.2	5.3±0.2

### Phytoplankton and Periphyton Biomass

**Figure 1:**
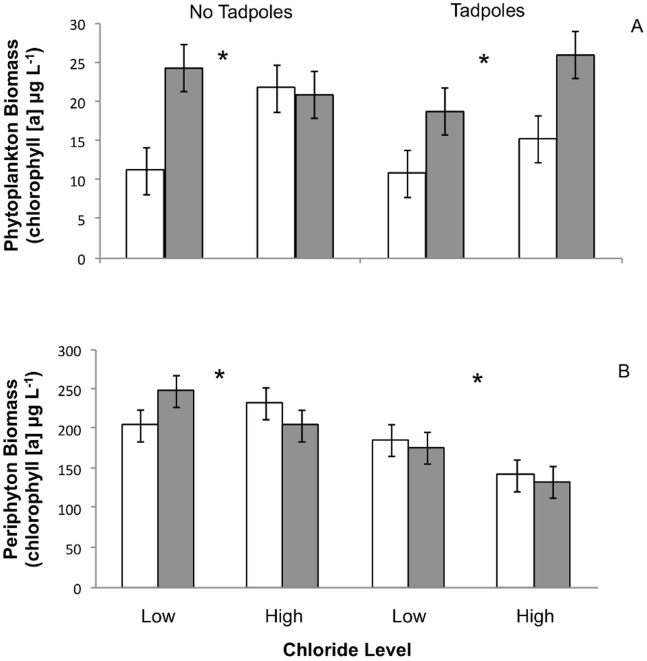
Mean phytoplankton (A) and periphyton (B) biomass (measured as chlorophyll [a] concentration in µg L^−1^) across chloride (low/high), tadpole (absent/present) and pond inoculum conductance level (low/high) treatments (n = 5 per treatment combination) over the duration of the study. Open bars represent low pond inoculum conductance levels and shaded bars represent high pond inoculum conductance levels. Asterisks indicate a statistically significant difference (p<0.05) between low and high chloride treatments using RMANOVA. Error bars represent standard error.

Phytoplankton was 29% more abundant in the high chloride treatment relative to the low chloride treatment (RMANOVA chloride main effect; Table 3; Table S2) and 53% greater in mesocosms receiving inoculum from the high conductance stormwater ponds as compared to ponds receiving inoculum from the low conductance stormwater ponds (RMANOVA inoculum main effect; Table 3, Fig. 1A). Neither the tadpole treatment (RMANOVA tadpole main effect; Table 3) nor sampling date (RMANOVA sampling date main effect; Table 3) had a significant main effect on phytoplankton biomass. There was a significant 3-way interactive effect of chloride treatment, inoculum source and sampling date on phytoplankton (RMANOVA chloride x inoculum x date effect F = 4.68_2,64_; *P* = 0.0126). On the first sampling date, phytoplankton biomass was much lower in the low chloride, low inoculum treatment relative to all other chloride and inoculum treatments (mean phytoplankton biomass: day 1 low chloride, low inoculum = 9.11 µg L^−1^; low chloride, high inoculum = 28.28 µg L^−1^; high chloride, high inoculum = 23.62 µg L^−1^; high chloride, low inoculum = 27.25 µg L^−1^). This trend continued throughout the second and third sampling dates for the low chloride, low inoculum treatment relative to the high chloride, high inoculum treatment (mean phytoplankton biomass: day 2 low chloride, low inoculum = 12.72 µg L^−1^; high chloride, high inoculum = 28.14 µg L^−1^; day 3 low chloride, low inoculum = 11.13 µg L^−1^; high chloride, high inoculum = 18.53 µg L^−1^).

**Table 3:** Repeated measures analysis of variance (RMAVNOVA) with compound symmetry covariance structure on mean values of log_10_ transformed phytoplankton abundance and periphyton abundance and analysis of variance (ANOVA) on mean values of log_10_ transformed total zooplankton density.

	Dependent Variable		
Effect	Phytoplankton	Periphyton	Total Zooplankton
**Chloride**			
* df*	1,32	1,32	1,32
* F*	4.31	4.19	20.56
* P*	**0.046**	**0.049**	**<0.0001**
**Tadpole**			
* df*	1,32	1,32	1,3
* F*	1.64	27.35	0.41
* P*	0.209	**<0.0001**	0.525
**Inoculum**			
* df*	1,32	1,32	1,32
* F*	14.6	0.04	0.40
* P*	**<0.001**	0.848	0.533
**Chloride x Tadpole**			
* df*	1,32	1,32	1,32
* F*	0.00	2.56	3.63
* P*	0.999	0.119	0.066
**Chloride x Inoculum**			
* df*	1,32	1,32	1,32
* F*	3.23	2.15	0.00
* P*	0.082	0.152	0.999
**Tadpole x Inoculum**			
* df*	1,32	1,32	1,32
* F*	0.03	0.72	0.01
* P*	0.862	0.401	0.937
**Chloride x Tadpole x Inoculum**			
* df*	1,32	1,32	1,32
* F*	1.34	0.43	1.67
* P*	0.256	0.517	0.206
**Sampling Date**			
* df*	2,64	2,64	−
* F*	1.9	10.69	−
* P*	0.1581	**<0.0001**	−

df = degrees of freedom; *F* = f-ratio; *P* = p-value; values in bold indicate statistical significance (p<0.05).

Periphyton biomass was 15% greater in the low chloride treatment than the high chloride treatment (RMANOVA chloride effect; Table 3; Table S3). In the mesocosms where tadpoles were present, periphyton biomass was 28% lower than in the mesocosms without tadpoles (RMANOVA tadpole effect; Table 3). Inoculum source had no significant effect (RMANOVA inoculum effect; Table 3, Fig. 1B). Periphyton biomass differed by sampling date (RMANOVA sampling date effect; Table 3) and there was a significant interaction between tadpole treatment and sampling date (RMANOVA tadpole x sampling date effect F = 7.67_2,64_; *P* = 0.001). Periphyton was least abundant mid-way through the study on June 30 (day 24), and this decline was most apparent in the mesocosms receiving high chloride, tadpoles and high conductance inoculum treatment (mean periphyton biomass: day 2 = 216.64±17.61 (SE) µg L^−1^; day 24 = 86.52±30.74 (SE) µg L^−1^; day 45 = 95.26±21.37 (SE) µg L^−1^day).

### Zooplankton Density & Community Composition

Total zooplankton density was significantly affected by the chloride treatment (ANOVA chloride effect; Table 3) but not by the tadpole (ANOVA tadpole effect; Table 3) or inoculum (ANOVA inoculum effect; Table 3) treatments. Total zooplankton density was 80% greater in the high chloride treatment relative to the low chloride treatment, largely due to an overwhelming abundance of rotifers in the high chloride treatment (see details on community composition below). None of the interactive effects between chloride, tadpole and inoculum treatments were significant with respect to total zooplankton density (ANOVA; Table 3).

**Figure 2:**
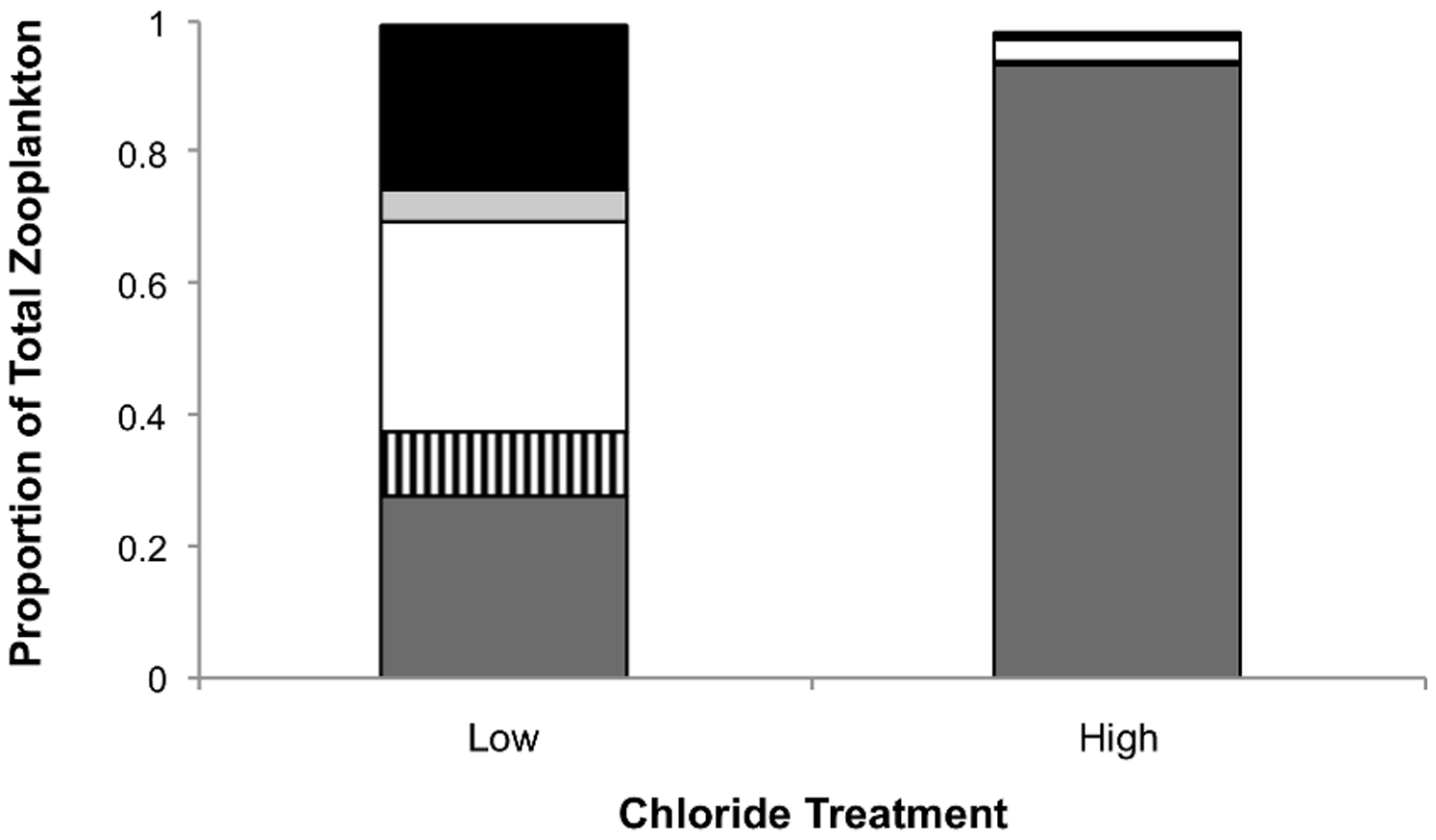
Zooplankton class or family as a proportion of total count across high and low chloride treatments. Dark gray bars represent the rotifer family Branchionidae, striped bars represent all other rotifers, open bars represent copepods, light gray bars represent ostracods and black bars represent cladocerans.

Across all treatments, we identified four rotifer families and 2 genera (family Brachionidae (genus *Brachionus* and *Keratella*), Synchaetidae, Notommatidae and Lecanidae), two copepod orders (Harpacticoid and Copepoid) and four families of cladocerans belonging to seven genera (family Sididae (genus *Diphanosoma*), Daphniidae (genus *Ceriodaphnia*, *Simocephalus*, *Scapholeberis* and *Daphnia*), Chydoridae (genus *Chydrous*) and Moinidae (genus *Moina*)) (Table S4). We did not identify ostracods or copepod nauplii (juveniles) beyond order. The zooplankton community in the high chloride treatment was dominated (93% of total zooplankton) by the rotifer family Brachionidae (3586 individuals belonging to family Brachionidae out of 3853 total zooplankton individuals counted in the high chloride treatment) (Fig. 2). Within this family, *Brachionus plicatilus*, a species common to salt and brackish waters [41], was the most abundant (data not shown). In contrast, the Brachionidae only accounted for 27% of total zooplankton community composition in the low chloride treatment (103 individuals belonging to family Brachionidae out of 379 total zooplankton individuals counted in the low chloride treatment). Ostracods, cladocerans, copepod adults and nauplii and other rotifers were much more abundant in the low chloride treatment relative to the high chloride treatment (Fig. 2).

The addition of chloride to the mesocosms resulted in a significant divergence in zooplankton community composition (chloride effect F_1,36_ = 22.5; *P* = 0.001). However, neither the inoculum source nor the tadpole treatment played a role in driving zooplankton community composition (inoculum effect F_1,36_ = 1.32; *P* = 0.204 and tadpole effect F_1,36_ = 1.04; *P* = 0.320). Similarly, there were no interactive effects between the chloride, tadpole and inoculum source treatments on the zooplankton community (all 2- and 3-way interactions *P*>0.05).

**Figure 3:**
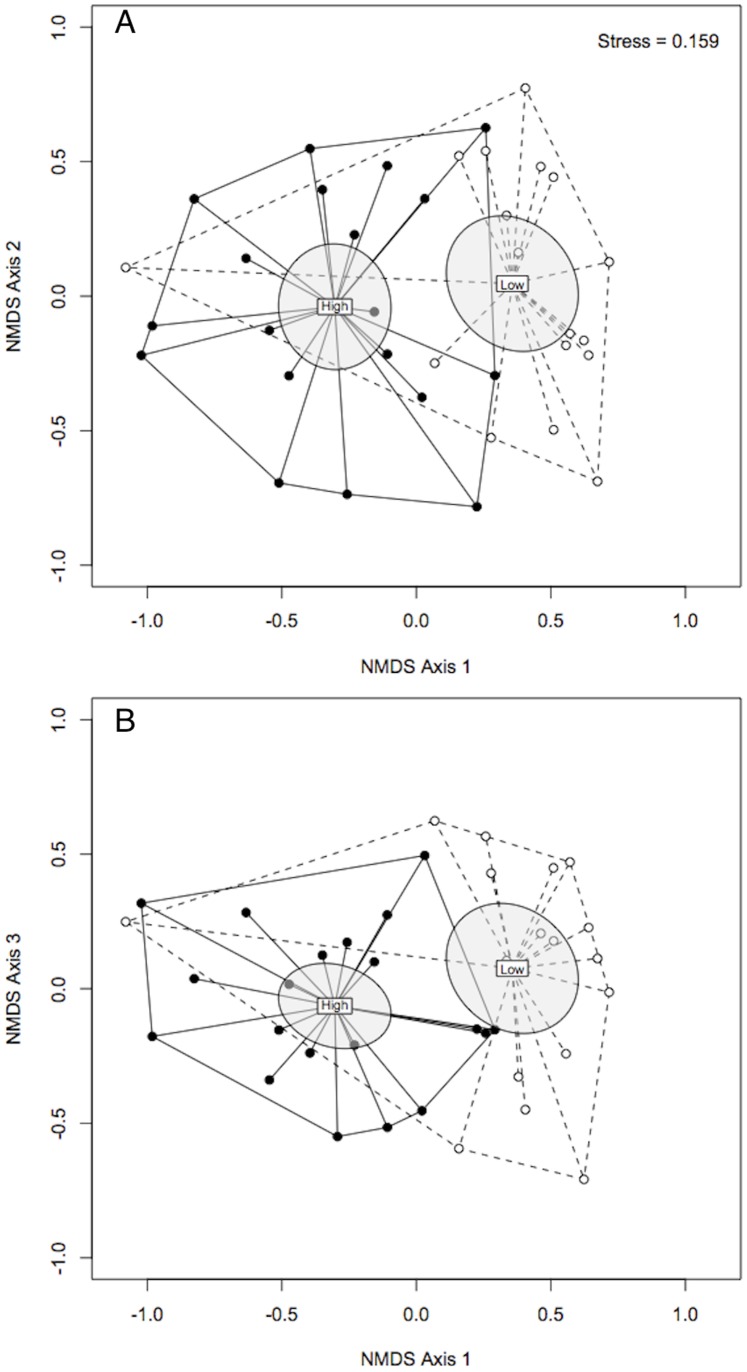
Non-metric multi-dimensional scaling (NMDS) results representing mesocosms receiving high (filled points) and low (open points) chloride. Points closer together indicate higher taxonomic similarity than those further apart. Centroids for each treatment reside under the “High” and “Low” labels. The shaded areas are the 95% confidence ellipses for the centroids. Each treatment group is bounded by the convex hull to aid in visualizing compositional spread about the centroids. The NMDS required three axes to produce a satisfactory stress (0.159). Therefore, axis 1 vs 2 (A) and 1 vs 3 (B) are portrayed. Note the scales of each are the same.

Zooplankton community composition within chloride treatments was much more similar (Bray-Curtis index High:High = 0.468; Low:Low = 0.666) than between chloride treatments (Bray-Curtis index values High:Low = 0.886). Furthermore, there was a significant difference in zooplankton species turnover between the high and low chloride treatments (F_1,35_ = 9.764; *P* = 0.003) suggesting high chloride served as a strong environmental constraint on taxon membership among mesocosms, and thus lower compositional turnover rate (Fig. 3). NMDS analysis results in 3-axes requiring an acceptable stress level of 0.159. Axis 1 is plotted against axes 2 and 3 separately, as visualizing axes 2 vs 3 is redundant.

### Tadpoles Survival, Mass at and Time to Metamorphosis

Average gray treefrog survival across all mesocosms was 90%. Neither chloride (Χ^2^ df = 1; *P* = 0.3175) nor inoculum (Χ^2^ df = 1; *P* = 0.8853) had an effect on survival. Mass at and time to metamorphosis were not affected by survival when used as a covariate, therefore survival was removed from the final analysis (Table S5). There was no significant effect of chloride, inoculum or the interaction between chloride and inoculum (chloride effect, inoculum effect, chloride x inoculum effect; Table 4) on days to metamorphosis.

**Table 4:** Analysis of variance (ANOVA) on mean values of gray treefrog (*Hyla versicolor*) days to metamorphosis and analysis of covariance (ANCOVA) on mean values of mass at metamorphosis with days to metamorphosis as covariate.

	Dependent Variable	
Effect	Days to Metamorphosis	Mass at Metamorphosis
* df*	1,16	1,12
**Chloride**		
* F*	0.06	5.83
* P*	0.808	**0.033**
**Inoculum**		
* F*	0.48	2.47
* P*	0.498	0.142
**Chloride x Inoculum**		
* F*	0.00	4.00
* P*	0.996	0.069
**Days to Metamorphosis**		
* F*	−	1.62
* P*	−	0.228
**Chloride × Days to Metamorphosis**		
* F*	−	5.4
* P*	−	**0.039**
**Inoculum × Days to Metamorphosis**		
* F*	−	2.46
* P*	−	0.143
**Chloride × Inoculum × Days to Metamorphosis**		
* F*	−	4.06
* P*	−	0.067

df = degrees of freedom; *F* = f-ratio; *P* = p-value; values in bold indicate statistical significance (p<0.05); values in bold indicate statistical significance (p<0.05).

When days to metamorphosis was used as a covariate, there was a significant effect of both chloride (chloride effect; Table 4) and the interaction between chloride and days to metamorphosis (chloride x days to metamorphosis effect; Table 4) on mass at metamorphosis. The higher chloride concentration in the mesocosms increased mass at metamorphosis by 8.5%. This chloride effect was only significant, however, for tadpoles experiencing a faster time to metamorphosis (25^th^ percentile = 46.65 days to metamorphosis). Among tadpoles experiencing faster time to metamorphosis, the average mass of metamorphs in the high chloride treatment was 326±18.2 (SE) mg and 270±18.0 (SE) mg in the low chloride treatment. This chloride effect was not significant for tadpoles reaching metamorphosis at or beyond the average number of days (average = 49.12 days; average mass in high chloride = 318±13.9 (SE) mg, low chloride = 295±13.8 (SE) mg; 75^th^ percentile = 51.63 days; average mass in high chloride = 309±16.2 (SE) mg, low chloride = 321±18.0 (SE) mg). The inoculum treatment had no significant main or interactive effects on gray treefrog mass at metamorphosis (Table 4).

## Discussion/Conclusions

Road salt run-off in urban areas has been documented as a pollutant source to freshwater ecosystems [17] and associated lethal and sublethal impacts to aquatic organisms continue to emerge [25], [29], [31]–[32], [42]–[46]. Our study highlights the potential for chloride salts to act as a significant environmental constraint on pond communities experiencing different specific conductance or road salt concentrations by limiting the aquatic community to more tolerant species. As predicted, the changes in phytoplankton biomass, zooplankton community composition and, to a smaller extent, the tadpole mass at metamorphosis results we obtained from our experimental mesocosm work does support the likelihood of environmental filtering in urban ponds by chloride salts. Furthermore, through identifying zooplankton taxa by colonist pool and across chloride treatments, our research emphasizes the role that tolerance to perceived environmental stressors plays in shaping impacted communities and in eliciting both direct and indirect effects within an aquatic food web. Chloride road salts, an emerging local environmental filter, may alter community composition and structure by presenting novel physiochemical conditions in typical freshwater habitats where species tolerances and thresholds are exceeded beyond their capacity for adaptation.

Our earlier surveys of stormwater ponds in the Red Run watershed, Owings Mills, Baltimore County, Maryland [29] revealed greater algal biomass in stormwater ponds across an increasing chloride gradient; this concurs with our current phytoplankton data. Relative to the low chloride treatment, phytoplankton biomass in the high chloride treatment was 29% greater in our experimental mesocosms. Furthermore, phytoplankton biomass was 53% greater in mesocosms that received the inoculum from higher conductance stormwater ponds in the Red Run watershed. Although we did not identify phytoplankton by class or species, we hypothesize that the phytoplankton communities developing in our mesocosms were filtered or sorted by their ability to acclimate to or tolerate high chloride loads. At concentrations even lower than those used in our study, salinity was identified as the main environmental driver of phytoplankton community composition and biomass in artificial sea salt-harvesting ponds in Tunisia [47]. Hypersaline ponds were dominated by phytoplankton class Chlorophyceae, a halophilic type of green algae. At its peak in 2003, phytoplankton biomass ranged from 2.5–12.5 times greater in a high salinity pond relative to the 3 lowest salinity ponds. Due to extreme saline conditions, phytoplankton communities in the hypersaline ponds were considered mature to the point of competitive exclusion [47]. Similarly, conductivity was reported as one of five major local environmental factors in predicting phytoplankton community structure in ponds in Belgium [11]. The conductivity range of these ponds was low relative to the conductivity we established in our experimental ponds. In considering community shifts in ponds with specific conductance values that closely resemble both our high and low chloride treatments, Olding [28] documented the dominance of marine and brackish water diatoms (e.g., *Caloneis amphibaena, Entomeoneis alata*) in a high conductance stormwater pond in Ontario, Canada while a lower conductance pond was dominated by nutrient tolerant taxa (e.g., *Euglena*). Pilkaityte et al. [48] found that sodium chloride shifts phytoplankton communities in favor of filamentous cyanobacteria at much higher salinities than those used in our study. Shifts in algal community structure and physiological parameters should be measured in future studies of algal resources experiencing salt stress. Given that species sorting toward tolerant taxa has been reported in a variety of studies at salinities both above and below those used here, is likely that salt stress contributed substantially to increases in phytoplankton biomass measured in our study.

Previous research exploring algal resources in stormwater ponds indicates that variation in tolerance to chloride inputs exists among species [27]–[28]. Differing from our predictions, we measured significantly greater periphyton biomass under low chloride conditions, regardless of inoculum source. These results do not indicate that chloride salts act as an environmental filter on periphyton in urban stormwater ponds, but likely reflect an observed interaction between phytoplankton and periphyton resources where abundant phytoplankton blooms can indirectly reduce periphyton biomass through shading effects [49]. Given that there was no significant interactive effect between chloride and tadpoles treatments, this precludes heavier tadpole grazing in high chloride treatments as a likely explanation for reduced periphyton biomass. To determine whether periphyton resources are declining as an indirect result of phytoplankton shading or through direct negative effects of elevated chloride and associated filtering, it would be necessary to study these algal resources in mesocosms independent of one another. However, this is logistically very difficult.

Filtering of zooplankton communities from salt loading in aquatic systems has been well documented in recent studies. Research on zooplankton assemblages from the Patuxent Wildlife Research Center (PWRC), Laurel, Maryland, a relatively pristine habitat, showed that elevated chloride levels (645 mg L^−1^ Cl^−^ = 2200 µS) were most harmful to adult copepods and cladocerans, however, copepod nauplii, ostracods and rotifers were unaffected [25]. Stormwater pond survey results showed that zooplankton densities differ across a chloride gradient with several taxa (ostracods, rotifers and cladocerans) colonizing medium conductance ponds (mean specific conductance ∼1150–2150 µS) at greater densities relative to high conductance ponds (mean specific conductance ∼ 6090–8370 µS; [29]). Zooplankton results from this study follow similar trends indicating that certain rotifers, particularly those belonging to family Branchionidae, proliferate under moderate conductance levels (∼3000 µS). Likewise, experimental research with wetland sediments from South Australia that were artificially flooded with a range of fresh to saline waters (300–15,000 mg L^−1^ salinity) resulted in substantially different zooplankton communities after a 21-day incubation period [50]. Sediments with higher natural salinities developed similar zooplankton communities to one another regardless of overlying water salinity, while the sediment with low natural salinity produced distinct communities with lower zooplankton species richness under more saline conditions. As confirmed by our study, more salt-tolerant zooplankton, such as the rotifer *Brachionus plicatilus* (Family Brachionidae), were most abundant in the microcosms containing high salinity sediments and water [50].

Tolerance thresholds to chloride salts differ greatly among zooplankton taxa and are known to serve as an important variable in determining zooplankton community composition and species richness. Previous research in southern France indicates that copepods are quite tolerant of high salinity conditions in temporary wetlands as 29% of cladoceran species recorded were found in high salinity waters [7]. Some cladoceran species are known as sensitive to the chloride concentrations similar to our low chloride treatment [51]–[52] while others are much more tolerant of elevated chloride levels [7], [53]–[55]. Bilkovic et al. [56] reported significant increases in total zooplankton densities in meso-oligohaline marshes after substantial periods of rainfall and subsequently lower salinities. While this increase in density was primarily due to proliferation of calanoid copepods under more freshwater conditions, cyclopoid copepods and cladoceran densities also increased. In two interrelated mesocosms studies, Nielson et al. [57]–[58] reported that with salinity increases (up to 15,000 mg L^−1^ sea salt), the developing zooplankton communities diverged greatly from the original freshwater species pool and ultimately resulted in domination by a small number of salt-tolerant species. As salinity levels in these artificial ponds were subsequently decreased (<2500 mg L^−1^ sea salt), zooplankton communities became increasingly similar with more taxa representative of freshwater systems. While freshwater zooplankton eggs can remain dormant under extremely elevated chloride levels and later successfully hatch, success of freshwater zooplankton is fully dependant on a return to lower salinity levels. Given substantial variability in tolerance to chloride salts among zooplankton, species composition is likely to differ within urban aquatic ecosystems experiencing perpetual road salt contamination and relative to more pristine habitats.

While there is strong evidence of direct effects of road salts in shaping the zooplankton community, indirect effects of salt on zooplankton and phytoplankton may also be prevalent in our study given that zooplankton are grazers of phytoplankton. Under elevated chloride conditions, tolerant phytoplankton species may have shaped the zooplankton community by providing species-specific preferred food resources. Conversely, chloride filtering among the zooplankton community may have indirectly resulted in dominance of non-preferred algal resources, thus accounting for increases in algal biomass under elevated salt conditions through a release in grazing pressure. A shift in community composition in stormwater ponds towards more tolerant taxa, whether propagated through direct or indirect effects, is likely given the temporal variation of chloride inputs and fluctuating hydrologic regime.

Much like zooplankton communities, research on the impacts of road salt deicers on tadpole grazers has revealed differential susceptibility among species. Early spring breeding Wood frog tadpoles (*Rana sylvatica*) have relatively low survival in the presence of road salts [23], [45] while later spring and summer breeding green frog tadpoles (*Rana clamitans*) have high survival rates [32]. Gray treefrogs (*Hyla versicolor*), the species used in our study, experience very low embryonic survival at chloride levels exceeding 1050 mg L^−1^ Cl- (∼ 3000 µS; [31]). However, gray treefrog larvae that persist during these early developmental stages do have high survival rates as they develop through metamorphosis in high salt concentrations [31]. Furthermore, these individuals reared under elevated chloride conditions experience faster time to and larger size at metamorphosis [25], [31]. We obtained gray treefrog embryos for these previous studies from PWRC which is a rather pristine and protected environment. Since the treefrogs used in the current study were obtained from stormwater detention ponds and presumably experience more consistent exposure to road salt inputs, we predicted that gray treefrogs living in or near stormwater detention ponds in urban areas would be better adapted to elevated chloride concentrations. As a result, changes in the time to and size at metamorphosis would only be apparent if elevated chloride inputs directly reduced the competing zooplankton population, thereby indirectly providing tadpole grazers with access to greater algal resources.

The urban gray treefrog population used in our study appears to be tolerant to elevated chloride road salt concentrations as common to stormwater detention ponds. Neither chloride nor inoculum treatment had direct effects on tadpole survival or mass at metamorphosis among gray treefrogs in the current study. Tadpoles in the high chloride treatment that metamorphosed quickly (within the 25^th^ percentile of days to metamorphosis) had a greater mass relative to those individuals at the average or greater than the average number of days to metamorphosis. This concurs with previous work [25], [31] and is unusual as frogs typically exhibit a trade-off between mass at and time to metamorphosis in which those that undergo metamorphosis early are usually smaller in size [59]. From previous research [25], we speculated that tadpoles developing under chloride salt stress had access to more abundant algal resources as a result of declines in zooplankton density. We cannot preclude the possibility of competitive release between tadpole and zooplankton grazers as a possible mechanism resulting in the greater size at metamorphosis in the high chloride treatment as seen in this study. Zooplankton belonging to family Brachonidae may not serve as strong competitors for gray treefrog tadpole preferred algal resources, but it is difficult to determine the magnitude of this interaction since our study was not designed with this species-specific competitive interaction in mind. Another possible explanation for increases size at metamorphosis is an osmoregulatory shift in tadpoles reared in the high chloride treatment. It is likely that chloride addition created a more isosmotic environment for the developing gray treefrog tadpoles, which are typically hyperosmotic to the surrounding aquatic environment [36]. Under isomotic conditions, tadpoles might redirect energy away from osmoregulation and redirect that energy towards feeding and growth. Given serious concerns about the long-term survival of amphibians in a human-dominated landscape [60], studies that evaluate road salt effects across generations and in combination with other biotic and abiotic stressors is strongly encouraged.

Environmental constraints, such as that due to chloride road salts, contribute substantially to the formation and development of urban aquatic communities and will play increasingly larger roles in developing urban ecosystems. Tolerance among urban pond dwelling organisms may be critical to their long-term survival in a constantly changing environment. Gaining a better understanding of tolerance thresholds and adaptive responses to pollutants in urban stormwater pond habitats will be important in evaluating potential changes in competitive and predatory interactions between and within trophic levels. As chloride salt loading in urban ponds fluctuates over time, dominant species within a community may oscillate in correspondence with environmental conditions and tolerance levels. Despite the fact that wetland acreage continues to decline across the globe [61]–[62] and urban ponds are compromised by high contaminant loads, these unique aquatic communities persist. With continued salinization of freshwaters, community composition may be shifted away from freshwater species and towards more brackish or marine species, thereby permanently altering the species pool in urban aquatic habitats.

## Supporting Information

Table S1 **Environmental variables across treatments and study days.**
(DOC)

Table S2 **Phytoplankton biomass on study days 5, 26 and 47.**
(DOC)

Table S3 **Periphyton biomass on study days 3, 24 and 45.**
(DOC)

Table S4 **Zooplankton count data.**
(XLS)

Table S5 **Tadpole metamorphosis data.**
(DOC)
