# Supplementary material for: Road Salts as Environmental Constraints in Urban Pond Food Webs
Source: PLoS One. 2014 Feb 26;9(2):e90168. doi: 10.1371/journal.pone.0090168 (PMC3935972; doi:10.1371/journal.pone.0090168)
Supplement: Table S1 — Environmental variables across treatments and study days. (DOC) [file pone.0090168.s001.doc]

| Table S1. Specific conductance (μS), temperature (Temp) (°C), dissolved oxygen (mg L^-1^) and nitrates (mg L^-1^) measured across 40 experimental mesocosms with chloride (high and low), tadpole (present or absent) and inoculum source (specific conductance = high or low) treatments (n = 5 per treatment combination). Specific conductance and temperature were measured three times on study days 0, 24 and 45 while dissolved oxygen and nitrates were measured once each (study day 24 and 45, respectively). | | | | | | | | | |
| --- | --- | --- | --- | --- | --- | --- | --- | --- | --- |
| Pond ID | Study Day | Chloride | Tadpoles | Inoculum | Specific Conductance | Temp | Dissolved Oxygen | | Nitrates |
| 1 | 0 | High | Present | High | 2993 | 21.7 | | - | - |
| 2 | 0 | Low | Absent | High | 674 | 22.1 | | - | - |
| 3 | 0 | Low | Present | High | 670 | 21.5 | | - | - |
| 4 | 0 | High | Present | Low | 3098 | 21.6 | | - | - |
| 5 | 0 | High | Absent | High | 3168 | 22 | | - | - |
| 6 | 0 | Low | Present | High | 669 | 21.8 | | - | - |
| 7 | 0 | Low | Present | Low | 648 | 21.6 | | - | - |
| 8 | 0 | Low | Absent | Low | 642 | 22.1 | | - | - |
| 9 | 0 | Low | Absent | High | 663 | 22.4 | | - | - |
| 10 | 0 | High | Absent | Low | 3055 | 21.7 | | - | - |
| 11 | 0 | High | Absent | High | 3025 | 21.8 | | - | - |
| 12 | 0 | High | Present | High | 2958 | 21.7 | | - | - |
| 13 | 0 | High | Absent | Low | 2861 | 22.1 | | - | - |
| 14 | 0 | Low | Present | Low | 667 | 22.4 | | - | - |
| 15 | 0 | Low | Present | High | 658 | 23 | | - | - |
| 16 | 0 | Low | Absent | High | 668 | 21.9 | | - | - |
| 17 | 0 | High | Absent | Low | 3042 | 22.2 | | - | - |
| 18 | 0 | High | Absent | High | 2945 | 21.6 | | - | - |
| 19 | 0 | High | Present | Low | 2835 | 21.9 | | - | - |
| 20 | 0 | Low | Absent | Low | 650 | 22.8 | | - | - |
| 21 | 0 | Low | Present | Low | 674 | 22.5 | | - | - |
| 22 | 0 | Low | Absent | Low | 670 | 22.2 | | - | - |
| 23 | 0 | High | Present | Low | 3129 | 21.8 | | - | - |
| 24 | 0 | High | Absent | High | 2834 | 22 | | - | - |
| 25 | 0 | High | Present | High | 3360 | 21.8 | | - | - |
| 26 | 0 | High | Absent | Low | 2984 | 21.7 | | - | - |
| 27 | 0 | High | Present | Low | 3100 | 22.3 | | - | - |
| 28 | 0 | Low | Present | Low | 683 | 21.9 | | - | - |
| 29 | 0 | Low | Present | High | 672 | 22.5 | | - | - |
| 30 | 0 | Low | Absent | Low | 691 | 22.3 | | - | - |
| 31 | 0 | Low | Absent | Low | 703 | 22.4 | | - | - |
| 32 | 0 | Low | Present | High | 703 | 22.2 | | - | - |
| 33 | 0 | Low | Absent | High | 705 | 22.2 | | - | - |
| 34 | 0 | High | Present | High | 2933 | 22.7 | | - | - |
| 35 | 0 | Low | Present | Low | 701 | 22.1 | | - | - |
| 36 | 0 | High | Present | Low | 2922 | 22.1 | | - | - |
| 37 | 0 | High | Absent | High | 2957 | 22 | | - | - |
| 38 | 0 | High | Present | High | 3100 | 22.4 | | - | - |
| 39 | 0 | High | Absent | Low | 3026 | 22.1 | | - | - |
| 40 | 0 | Low | Absent | High | 699 | 22.5 | | - | - |
| 1 | 24 | High | Present | High | 2827 | 25.3 | | 4.9 | - |
| 2 | 24 | Low | Absent | High | 538 | 25.5 | | 3.94 | - |
| 3 | 24 | Low | Present | High | 533 | 25.6 | | 5.43 | - |
| 4 | 24 | High | Present | Low | 2724 | 25.6 | | 4.39 | - |
| 5 | 24 | High | Absent | High | 2863 | 25.5 | | 6.01 | - |
| 6 | 24 | Low | Present | High | 541 | 25.4 | | 4.55 | - |
| 7 | 24 | Low | Present | Low | 514 | 24.8 | | 3.69 | - |
| 8 | 24 | Low | Absent | Low | 513 | 26 | | 6.16 | - |
| 9 | 24 | Low | Absent | High | 533 | 25.8 | | 5.04 | - |
| 10 | 24 | High | Absent | Low | 2699 | 25.5 | | 4.7 | - |
| 11 | 24 | High | Absent | High | 2736 | 25.6 | | 5.6 | - |
| 12 | 24 | High | Present | High | 2730 | 25.6 | | 4.98 | - |
| 13 | 24 | High | Absent | Low | 2708 | 25.6 | | 5.37 | - |
| 14 | 24 | Low | Present | Low | 517 | 25.6 | | 3.27 | - |
| 15 | 24 | Low | Present | High | 532 | 25.6 | | 4.35 | - |
| 16 | 24 | Low | Absent | High | 532 | 25.5 | | 6.35 | - |
| 17 | 24 | High | Absent | Low | 2743 | 25.6 | | 6.8 | - |
| 18 | 24 | High | Absent | High | 2757 | 25.7 | | 4.38 | - |
| 19 | 24 | High | Present | Low | 2768 | 25.5 | | 4.52 | - |
| 20 | 24 | Low | Absent | Low | 522 | 25.5 | | 5.6 | - |
| 21 | 24 | Low | Present | Low | 536 | 25.3 | | 5 | - |
| 22 | 24 | Low | Absent | Low | 538 | 25.4 | | 6.09 | - |
| 23 | 24 | High | Present | Low | 2824 | 25.4 | | 4.58 | - |
| 24 | 24 | High | Absent | High | 2904 | 25.4 | | 6.12 | - |
| 25 | 24 | High | Present | High | 2915 | 25.5 | | 6.05 | - |
| 26 | 24 | High | Absent | Low | 2849 | 25.6 | | 5.85 | - |
| 27 | 24 | High | Present | Low | 2877 | 25.4 | | 5 | - |
| 28 | 24 | Low | Present | Low | 538 | 25.4 | | 4.61 | - |
| 29 | 24 | Low | Present | High | 542 | 25.4 | | 5.07 | - |
| 30 | 24 | Low | Absent | Low | 534 | 25.5 | | 5.76 | - |
| 31 | 24 | Low | Absent | Low | 549 | 25.7 | | 5.89 | - |
| 32 | 24 | Low | Present | High | 559 | 25.7 | | 5.09 | - |
| 33 | 24 | Low | Absent | High | 564 | 25.4 | | 5.64 | - |
| 34 | 24 | High | Present | High | 2896 | 25.5 | | 5.67 | - |
| 35 | 24 | Low | Present | Low | 534 | 25.3 | | 6.57 | - |
| 36 | 24 | High | Present | Low | 2874 | 25.5 | | 5.18 | - |
| 37 | 24 | High | Absent | High | 2895 | 25.7 | | 5.16 | - |
| 38 | 24 | High | Present | High | 2907 | 25.5 | | 6.5 | - |
| 39 | 24 | High | Absent | Low | 2816 | 25.6 | | 4.56 | - |
| 40 | 24 | Low | Absent | High | 560 | 25.5 | | 6.14 | - |
| 1 | 45 | High | Present | High | 2709 | 25.5 | | - | 0.000 |
| 2 | 45 | Low | Absent | High | 509 | 25.6 | | - | 0.001 |
| 3 | 45 | Low | Present | High | 513 | 27 | | - | 0.001 |
| 4 | 45 | High | Present | Low | 2596 | 25.5 | | - | 0.004 |
| 5 | 45 | High | Absent | High | 2774 | 25.7 | | - | 0.000 |
| 6 | 45 | Low | Present | High | 522 | 26.5 | | - | 0.007 |
| 7 | 45 | Low | Present | Low | 495 | 26.6 | | - | 0.009 |
| 8 | 45 | Low | Absent | Low | 491 | 26.7 | | - | 0.014 |
| 9 | 45 | Low | Absent | High | 511 | 27 | | - | 0.000 |
| 10 | 45 | High | Absent | Low | 2581 | 25.9 | | - | 0.005 |
| 11 | 45 | High | Absent | High | 2631 | 25.4 | | - | 0.000 |
| 12 | 45 | High | Present | High | 2622 | 26.4 | | - | 0.007 |
| 13 | 45 | High | Absent | Low | 2599 | 26.2 | | - | 0.003 |
| 14 | 45 | Low | Present | Low | 501 | 26.3 | | - | 0.005 |
| 15 | 45 | Low | Present | High | 514 | 26.8 | | - | 0.005 |
| 16 | 45 | Low | Absent | High | 508 | 26.7 | | - | 0.001 |
| 17 | 45 | High | Absent | Low | 2642 | 24.9 | | - | 0.000 |
| 18 | 45 | High | Absent | High | 2644 | 25.4 | | - | 0.000 |
| 19 | 45 | High | Present | Low | 2662 | 25.2 | | - | 0.001 |
| 20 | 45 | Low | Absent | Low | 499 | 26.4 | | - | 0.007 |
| 21 | 45 | Low | Present | Low | 513 | 26 | | - | 0.007 |
| 22 | 45 | Low | Absent | Low | 513 | 25.4 | | - | 0.007 |
| 23 | 45 | High | Present | Low | 2698 | 26.1 | | - | 0.006 |
| 24 | 45 | High | Absent | High | 2792 | 25.8 | | - | 0.005 |
| 25 | 45 | High | Present | High | 2791 | 25.2 | | - | 0.000 |
| 26 | 45 | High | Absent | Low | 2754 | 26.2 | | - | 0.001 |
| 27 | 45 | High | Present | Low | 2761 | 26.2 | | - | 0.001 |
| 28 | 45 | Low | Present | Low | 516 | 25.5 | | - | 0.001 |
| 29 | 45 | Low | Present | High | 514 | 25.5 | | - | 0.000 |
| 30 | 45 | Low | Absent | Low | 512 | 26.3 | | - | 0.005 |
| 31 | 45 | Low | Absent | Low | 530 | 26.5 | | - | 0.001 |
| 32 | 45 | Low | Present | High | 535 | 26.9 | | - | 0.005 |
| 33 | 45 | Low | Absent | High | 535 | 25.5 | | - | 0.001 |
| 34 | 45 | High | Present | High | 2769 | 25.9 | | - | 0.005 |
| 35 | 45 | Low | Present | Low | 506 | 25.6 | | - | 0.005 |
| 36 | 45 | High | Present | Low | 2763 | 26.3 | | - | 0.019 |
| 37 | 45 | High | Absent | High | 2790 | 26.5 | | - | 0.001 |
| 38 | 45 | High | Present | High | 2792 | 26.7 | | - | 0.001 |
| 39 | 45 | High | Absent | Low | 2739 | 25.4 | | - | 0.010 |
| 40 | 45 | Low | Absent | High | 540 | 25.7 | | - | 0.000 |
