# Supplementary material for: Road Salts as Environmental Constraints in Urban Pond Food Webs
Source: PLoS One. 2014 Feb 26;9(2):e90168. doi: 10.1371/journal.pone.0090168 (PMC3935972; doi:10.1371/journal.pone.0090168)
Supplement: Table S2 — Phytoplankton biomass on study days 5, 26 and 47. (DOC) [file pone.0090168.s002.doc]

| Table S2: Phytoplankton biomass (chlorophyll [a] μg L^-1^) sample estimates and mesocosm means across 40 experimental mesocosms on study days 5, 26 and 47 with chloride (high or low), tadpole (present or absent) and inoculum source (specific conductance = high or low) treatments. Blank samples were composed of acetone only. | | | | | | | | |
| --- | --- | --- | --- | --- | --- | --- | --- | --- |
| Pond ID | Chloride | Tadpoles | Inoculum | Date | Sample 1 | Sample 2 | Sample 3 | Mean |
| 1 | High | Present | High | 5 | 37.4 | 36.7 | 36.8 | 37.0 |
| 2 | Low | Absent | High | 5 | 16.9 | 17.2 | 17.2 | 17.1 |
| 3 | Low | Present | High | 5 | 10.2 | 10.1 | 10.4 | 10.2 |
| 4 | High | Present | Low | 5 | 19.3 | 19.3 | 19.4 | 19.3 |
| 5 | High | Absent | High | 5 | 36.2 | 36.7 | 36.7 | 36.5 |
| 6 | Low | Present | High | 5 | 9.9 | 10.3 | 9.9 | 10.1 |
| 7 | Low | Present | Low | 5 | 5.3 | 5.3 | 5.4 | 5.4 |
| 8 | Low | Absent | Low | 5 | 15.3 | 15.3 | 15.4 | 15.4 |
| 9 | Low | Absent | High | 5 | 24.0 | 23.6 | 23.8 | 23.8 |
| 10 | High | Absent | Low | 5 | 30.2 | 30.2 | 30.5 | 30.3 |
| 11 | High | Absent | High | 5 | 10.5 | 10.6 | 10.5 | 10.5 |
| 12 | High | Present | High | 5 | 18.7 | 18.6 | 18.6 | 18.6 |
| 13 | High | Absent | Low | 5 | 45.5 | 45.4 | 45.5 | 45.5 |
| 14 | Low | Present | Low | 5 | 6.5 | 7.1 | 6.7 | 6.8 |
| 15 | Low | Present | High | 5 | 42.8 | 42.8 | 42.4 | 42.6 |
| 16 | Low | Absent | High | 5 | 63.5 | 62.9 | 63.1 | 63.2 |
| 17 | High | Absent | Low | 5 | 38.0 | 38.2 | 37.8 | 38.0 |
| 18 | High | Absent | High | 5 | 13.5 | 13.8 | 13.5 | 13.6 |
| 19 | High | Present | Low | 5 | 7.2 | 7.2 | 7.1 | 7.2 |
| 20 | Low | Absent | Low | 5 | 10.6 | 10.6 | 10.5 | 10.5 |
| 21 | Low | Present | Low | 5 | 6.7 | 6.8 | 6.7 | 6.7 |
| 22 | Low | Absent | Low | 5 | 4.0 | 4.1 | 4.1 | 4.0 |
| 23 | High | Present | Low | 5 | 40.1 | 40.4 | 40.3 | 40.3 |
| 24 | High | Absent | High | 5 | 22.2 | 22.3 | 22.3 | 22.3 |
| 25 | High | Present | High | 5 | 27.6 | 27.6 | 27.4 | 27.5 |
| 26 | High | Absent | Low | 5 | 50.7 | 50.2 | 50.5 | 50.5 |
| 27 | High | Present | Low | 5 | 11.5 | 11.9 | 11.9 | 11.8 |
| 28 | Low | Present | Low | 5 | 13.7 | 13.7 | 13.7 | 13.7 |
| 29 | Low | Present | High | 5 | 34.8 | 34.4 | 34.5 | 34.6 |
| 30 | Low | Absent | Low | 5 | 5.2 | 5.1 | 5.0 | 5.1 |
| 31 | Low | Absent | Low | 5 | 9.7 | 9.8 | 9.8 | 9.7 |
| 32 | Low | Present | High | 5 | 17.7 | 17.6 | 17.5 | 17.6 |
| 33 | Low | Absent | High | 5 | 31.3 | 31.0 | 31.0 | 31.1 |
| 34 | High | Present | High | 5 | 20.7 | 20.6 | 20.6 | 20.6 |
| 35 | Low | Present | Low | 5 | 13.9 | 13.9 | 13.8 | 13.8 |
| 36 | High | Present | Low | 5 | 25.7 | 25.5 | 25.6 | 25.6 |
| 37 | High | Absent | High | 5 | 21.7 | 21.8 | 21.6 | 21.7 |
| 38 | High | Present | High | 5 | 27.8 | 28.3 | 28.1 | 28.0 |
| 39 | High | Absent | Low | 5 | 4.1 | 4.2 | 4.3 | 4.2 |
| 40 | Low | Absent | High | 5 | 32.8 | 32.5 | 32.8 | 32.7 |
| Blank |  |  |  | 5 | 0.0 | 0.0 | 0.0 | 0.0 |
| 1 | High | Present | High | 26 | 14.5 | 14.4 | 14.5 | 14.5 |
| 2 | Low | Absent | High | 26 | 14.6 | 14.6 | 14.4 | 14.6 |
| 3 | Low | Present | High | 26 | 9.6 | 9.6 | 9.6 | 9.6 |
| 4 | High | Present | Low | 26 | 5.7 | 5.7 | 5.7 | 5.7 |
| 5 | High | Absent | High | 26 | 31.6 | 31.6 | 31.3 | 31.5 |
| 6 | Low | Present | High | 26 | 10.0 | 10.4 | 9.8 | 10.0 |
| 7 | Low | Present | Low | 26 | 12.2 | 12.2 | 12.1 | 12.2 |
| 8 | Low | Absent | Low | 26 | 10.0 | 10.0 | 10.0 | 10.0 |
| 9 | Low | Absent | High | 26 | 11.7 | 11.6 | 11.6 | 11.6 |
| 10 | High | Absent | Low | 26 | 20.8 | 20.7 | 20.6 | 20.7 |
| 11 | High | Absent | High | 26 | 10.4 | 10.5 | 10.4 | 10.4 |
| 12 | High | Present | High | 26 | 11.9 | 11.8 | 11.8 | 11.9 |
| 13 | High | Absent | Low | 26 | 12.7 | 12.6 | 12.5 | 12.6 |
| 14 | Low | Present | Low | 26 | 8.8 | 8.8 | 8.8 | 8.8 |
| 15 | Low | Present | High | 26 | 12.0 | 12.2 | 12.0 | 12.0 |
| 16 | Low | Absent | High | 26 | 16.0 | 16.0 | 15.9 | 16.0 |
| 17 | High | Absent | Low | 26 | 13.7 | 13.7 | 13.7 | 13.7 |
| 18 | High | Absent | High | 26 | 11.8 | 11.7 | 11.8 | 11.8 |
| 19 | High | Present | Low | 26 | 15.0 | 14.9 | 15.0 | 15.0 |
| 20 | Low | Absent | Low | 26 | 10.1 | 10.1 | 10.1 | 10.1 |
| 21 | Low | Present | Low | 26 | 12.6 | 12.7 | 12.7 | 12.7 |
| 22 | Low | Absent | Low | 26 | 11.7 | 11.7 | 11.5 | 11.6 |
| 23 | High | Present | Low | 26 | 8.8 | 8.8 | 8.9 | 8.8 |
| 24 | High | Absent | High | 26 | 14.2 | 14.2 | 14.2 | 14.2 |
| 25 | High | Present | High | 26 | 10.8 | 10.8 | 10.8 | 10.8 |
| 26 | High | Absent | Low | 26 | 12.6 | 12.5 | 12.5 | 12.6 |
| 27 | High | Present | Low | 26 | 11.1 | 11.0 | 11.0 | 11.0 |
| 28 | Low | Present | Low | 26 | 17.7 | 18.0 | 17.9 | 17.9 |
| 29 | Low | Present | High | 26 | 20.7 | 20.6 | 20.5 | 20.6 |
| 30 | Low | Absent | Low | 26 | 8.6 | 8.8 | 8.6 | 8.7 |
| 31 | Low | Absent | Low | 26 | 16.0 | 16.0 | 15.8 | 15.9 |
| 32 | Low | Present | High | 26 | 22.5 | 22.5 | 22.3 | 22.4 |
| 33 | Low | Absent | High | 26 | 33.4 | 33.6 | 33.6 | 33.5 |
| 34 | High | Present | High | 26 | 108.5 | 108.6 | 107.1 | 108.1 |
| 35 | Low | Present | Low | 26 | 19.4 | 19.7 | 19.4 | 19.5 |
| 36 | High | Present | Low | 26 | 7.8 | 7.9 | 7.8 | 7.8 |
| 37 | High | Absent | High | 26 | 31.2 | 30.9 | 31.1 | 31.0 |
| 38 | High | Present | High | 26 | 37.3 | 37.4 | 37.1 | 37.3 |
| 39 | High | Absent | Low | 26 | 9.1 | 8.8 | 9.1 | 9.0 |
| 40 | Low | Absent | High | 26 | 15.4 | 15.3 | 15.2 | 15.3 |
| Blank |  |  |  | 26 | 0.0 | 0.0 | 0.0 | 0.0 |
| 1 | High | Present | High | 47 | 3.2 | 3.2 | 3.2 | 3.2 |
| 2 | Low | Absent | High | 47 | 5.8 | 5.8 | 5.6 | 5.7 |
| 3 | Low | Present | High | 47 | 21.5 | 21.3 | 21.2 | 21.3 |
| 4 | High | Present | Low | 47 | 12.8 | 12.7 | 12.8 | 12.8 |
| 5 | High | Absent | High | 47 | 31.1 | 31.1 | 30.9 | 31.0 |
| 6 | Low | Present | High | 47 | 30.9 | 30.4 | 30.7 | 30.7 |
| 7 | Low | Present | Low | 47 | 9.9 | 9.8 | 9.9 | 9.8 |
| 8 | Low | Absent | Low | 47 | 18.3 | 18.0 | 18.0 | 18.1 |
| 9 | Low | Absent | High | 47 | 28.9 | 28.6 | 28.1 | 28.5 |
| 10 | High | Absent | Low | 47 | 18.0 | 18.0 | 18.0 | 18.0 |
| 11 | High | Absent | High | 47 | 14.7 | 14.4 | 14.5 | 14.5 |
| 12 | High | Present | High | 47 | 33.4 | 33.3 | 33.1 | 33.3 |
| 13 | High | Absent | Low | 47 | 7.4 | 7.3 | 7.3 | 7.3 |
| 14 | Low | Present | Low | 47 | 7.9 | 7.8 | 7.8 | 7.8 |
| 15 | Low | Present | High | 47 | 6.9 | 6.9 | 6.9 | 6.9 |
| 16 | Low | Absent | High | 47 | 24.3 | 24.2 | 24.4 | 24.3 |
| 17 | High | Absent | Low | 47 | 25.0 | 5.9 | 22.3 | 17.7 |
| 18 | High | Absent | High | 47 | 14.6 | 14.2 | 14.3 | 14.4 |
| 19 | High | Present | Low | 47 | 18.5 | 18.6 | 18.5 | 18.6 |
| 20 | Low | Absent | Low | 47 | 7.1 | 7.0 | 7.2 | 7.1 |
| 21 | Low | Present | Low | 47 | 8.3 | 8.2 | 8.1 | 8.2 |
| 22 | Low | Absent | Low | 47 | 9.5 | 9.7 | 9.5 | 9.6 |
| 23 | High | Present | Low | 47 | 16.1 | 3.6 | 15.1 | 11.6 |
| 24 | High | Absent | High | 47 | 18.4 | 18.4 | 18.3 | 18.4 |
| 25 | High | Present | High | 47 | 8.8 | 8.7 | 8.7 | 8.7 |
| 26 | High | Absent | Low | 47 | 23.3 | 23.1 | 23.1 | 23.2 |
| 27 | High | Present | Low | 47 | 12.1 | 12.3 | 12.5 | 12.3 |
| 28 | Low | Present | Low | 47 | 4.8 | 5.0 | 5.3 | 5.1 |
| 29 | Low | Present | High | 47 | 14.4 | 14.2 | 14.2 | 14.3 |
| 30 | Low | Absent | Low | 47 | 13.2 | 12.8 | 12.9 | 13.0 |
| 31 | Low | Absent | Low | 47 | 18.1 | 18.2 | 18.1 | 18.2 |
| 32 | Low | Present | High | 47 | 18.7 | 18.7 | 18.8 | 18.7 |
| 33 | Low | Absent | High | 47 | 33.7 | 32.9 | 33.2 | 33.3 |
| 34 | High | Present | High | 47 | 14.3 | 14.3 | 14.3 | 14.3 |
| 35 | Low | Present | Low | 47 | 13.6 | 13.5 | 13.4 | 13.5 |
| 36 | High | Present | Low | 47 | 20.8 | 20.4 | 20.5 | 20.5 |
| 37 | High | Absent | High | 47 | 31.6 | 31.6 | 31.5 | 31.6 |
| 38 | High | Present | High | 47 | 16.4 | 16.0 | 15.8 | 16.1 |
| 39 | High | Absent | Low | 47 | 22.0 | 22.1 | 21.7 | 21.9 |
| 40 | Low | Absent | High | 47 | 14.1 | 14.0 | 14.1 | 14.1 |
| Blank |  |  |  | 47 | 0.0 | 0.0 | 0.0 | 0.0 |
