# Supplementary material for: Road Salts as Environmental Constraints in Urban Pond Food Webs
Source: PLoS One. 2014 Feb 26;9(2):e90168. doi: 10.1371/journal.pone.0090168 (PMC3935972; doi:10.1371/journal.pone.0090168)
Supplement: Table S3 — Periphyton biomass on study days 3, 24 and 45. (DOC) [file pone.0090168.s003.doc]

| Table S3: Periphtyon biomass (chlorophyll [a] μg L^-1^) sample estimates and mesocosm means across 40 experimental mesocosms on study days 3, 24 and 45 with chloride (high or low), tadpole (present or absent) and inoculum source (specific conductance = high or low) treatments. Blank samples were composed of acetone only. | | | | | | | | |
| --- | --- | --- | --- | --- | --- | --- | --- | --- |
| Pond ID | Chloride | Tadpoles | Inoculum | Study Day | Sample 1 | Sample 2 | Sample 3 | Mean |
| 1 | High | Present | High | 3 | 205.9 | 212.5 | 208.5 | 209.0 |
| 2 | Low | Absent | High | 3 | 202.5 | 201.9 | 201.6 | 202.0 |
| 3 | Low | Present | High | 3 | 245.4 | 245.6 | 245.8 | 245.6 |
| 4 | High | Present | Low | 3 | 259.5 | 254.0 | 265.2 | 259.6 |
| 5 | High | Absent | High | 3 | 212.2 | 216.9 | 220.6 | 216.6 |
| 6 | Low | Present | High | 3 | 157.3 | 158.8 | 166.4 | 160.9 |
| 7 | Low | Present | Low | 3 | 155.0 | 158.1 | 159.1 | 157.4 |
| 8 | Low | Absent | Low | 3 | 156.1 | 155.6 | 156.5 | 156.0 |
| 9 | Low | Absent | High | 3 | 258.4 | 264.5 | 261.0 | 261.3 |
| 10 | High | Absent | Low | 3 | 276.3 | 266.4 | 271.3 | 271.3 |
| 11 | High | Absent | High | 3 | 309.9 | 309.8 | 304.5 | 308.0 |
| 12 | High | Present | High | 3 | 232.8 | 233.1 | 231.5 | 232.4 |
| 13 | High | Absent | Low | 3 | 176.3 | 174.8 | 175.8 | 175.6 |
| 14 | Low | Present | Low | 3 | 167.4 | 165.8 | 162.5 | 165.2 |
| 15 | Low | Present | High | 3 | 308.3 | 304.5 | 309.9 | 307.6 |
| 16 | Low | Absent | High | 3 | 259.3 | 259.8 | 260.9 | 260.0 |
| 17 | High | Absent | Low | 3 | 175.5 | 190.9 | 191.4 | 185.9 |
| 18 | High | Absent | High | 3 | 181.3 | 179.7 | 180.3 | 180.5 |
| 19 | High | Present | Low | 3 | 104.8 | 112.6 | 142.1 | 119.9 |
| 20 | Low | Absent | Low | 3 | 190.9 | 188.4 | 191.2 | 190.1 |
| 21 | Low | Present | Low | 3 | 264.6 | 263.6 | 262.5 | 263.6 |
| 22 | Low | Absent | Low | 3 | 245.0 | 244.4 | 241.9 | 243.8 |
| 23 | High | Present | Low | 3 | 269.0 | 263.9 | 268.9 | 267.2 |
| 24 | High | Absent | High | 3 | 173.0 | 170.9 | 176.9 | 173.6 |
| 25 | High | Present | High | 3 | 179.5 | 184.0 | 181.8 | 181.8 |
| 26 | High | Absent | Low | 3 | 243.2 | 255.7 | 254.6 | 251.1 |
| 27 | High | Present | Low | 3 | 145.5 | 146.1 | 148.9 | 146.8 |
| 28 | Low | Present | Low | 3 | 200.4 | 195.0 | 184.1 | 193.2 |
| 29 | Low | Present | High | 3 | 277.9 | 276.9 | 275.2 | 276.7 |
| 30 | Low | Absent | Low | 3 | 195.9 | 195.5 | 195.6 | 195.7 |
| 31 | Low | Absent | Low | 3 | 133.1 | 136.3 | 136.1 | 135.2 |
| 32 | Low | Present | High | 3 | 236.4 | 241.5 | 238.8 | 238.9 |
| 33 | Low | Absent | High | 3 | 214.1 | 191.1 | 196.7 | 200.6 |
| 34 | High | Present | High | 3 | 282.9 | 270.6 | 279.7 | 277.7 |
| 35 | Low | Present | Low | 3 | 225.9 | 229.7 | 228.1 | 227.9 |
| 36 | High | Present | Low | 3 | 220.4 | 218.4 | 223.6 | 220.8 |
| 37 | High | Absent | High | 3 | 247.9 | 257.5 | 243.6 | 249.7 |
| 38 | High | Present | High | 3 | 181.3 | 182.1 | 194.2 | 185.9 |
| 39 | High | Absent | Low | 3 | 133.1 | 136.4 | 134.6 | 134.7 |
| 40 | Low | Absent | High | 3 | 308.7 | 311.6 | 311.7 | 310.7 |
| Blank |  |  |  | 3 | 0.7 | 0.7 | 0.7 | 0.7 |
| 1 | High | Present | High | 24 | 88.6 | 87.2 | 86.6 | 87.5 |
| 2 | Low | Absent | High | 24 | 138.2 | 136.9 | 136.8 | 137.3 |
| 3 | Low | Present | High | 24 | 122.4 | 121.5 | 121.5 | 121.8 |
| 4 | High | Present | Low | 24 | 108.0 | 106.5 | 107.2 | 107.2 |
| 5 | High | Absent | High | 24 | 181.8 | 182.3 | 181.6 | 181.9 |
| 6 | Low | Present | High | 24 | 223.0 | 224.8 | 223.6 | 223.8 |
| 7 | Low | Present | Low | 24 | 123.4 | 124.7 | 125.4 | 124.5 |
| 8 | Low | Absent | Low | 24 | 384.7 | 385.2 | 382.9 | 384.3 |
| 9 | Low | Absent | High | 24 | 175.1 | 179.0 | 179.8 | 178.0 |
| 10 | High | Absent | Low | 24 | 176.0 | 177.4 | 174.6 | 176.0 |
| 11 | High | Absent | High | 24 | 235.4 | 236.7 | 235.4 | 235.8 |
| 12 | High | Present | High | 24 | 167.9 | 169.2 | 166.1 | 167.7 |
| 13 | High | Absent | Low | 24 | 132.1 | 131.8 | 131.2 | 131.7 |
| 14 | Low | Present | Low | 24 | 45.4 | 46.4 | 46.0 | 46.0 |
| 15 | Low | Present | High | 24 | 71.5 | 71.9 | 71.2 | 71.5 |
| 16 | Low | Absent | High | 24 | 200.3 | 198.1 | 194.3 | 197.6 |
| 17 | High | Absent | Low | 24 | 273.7 | 273.1 | 280.6 | 275.8 |
| 18 | High | Absent | High | 24 | 153.2 | 154.5 | 152.8 | 153.5 |
| 19 | High | Present | Low | 24 | 122.1 | 122.6 | 122.0 | 122.2 |
| 20 | Low | Absent | Low | 24 | 96.7 | 96.0 | 96.1 | 96.3 |
| 21 | Low | Present | Low | 24 | 168.5 | 166.8 | 166.3 | 167.2 |
| 22 | Low | Absent | Low | 24 | 222.6 | 223.7 | 222.6 | 223.0 |
| 23 | High | Present | Low | 24 | 92.3 | 93.2 | 93.6 | 93.0 |
| 24 | High | Absent | High | 24 | 98.0 | 96.7 | 97.2 | 97.3 |
| 25 | High | Present | High | 24 | 93.4 | 93.1 | 93.2 | 93.2 |
| 26 | High | Absent | Low | 24 | 366.5 | 366.0 | 366.3 | 366.3 |
| 27 | High | Present | Low | 24 | 72.2 | 75.1 | 74.8 | 74.0 |
| 28 | Low | Present | Low | 24 | 82.5 | 82.2 | 82.6 | 82.4 |
| 29 | Low | Present | High | 24 | 38.8 | 39.9 | 39.1 | 39.3 |
| 30 | Low | Absent | Low | 24 | 192.3 | 191.6 | 190.4 | 191.4 |
| 31 | Low | Absent | Low | 24 | 268.3 | 267.7 | 268.3 | 268.1 |
| 32 | Low | Present | High | 24 | 133.9 | 136.1 | 135.4 | 135.2 |
| 33 | Low | Absent | High | 24 | 288.4 | 285.6 | 285.5 | 286.5 |
| 34 | High | Present | High | 24 | 53.3 | 56.7 | 53.8 | 54.6 |
| 35 | Low | Present | Low | 24 | 372.7 | 372.0 | 371.6 | 372.1 |
| 36 | High | Present | Low | 24 | 151.9 | 154.1 | 152.9 | 153.0 |
| 37 | High | Absent | High | 24 | 186.1 | 186.5 | 185.0 | 185.8 |
| 38 | High | Present | High | 24 | 30.6 | 30.0 | 28.3 | 29.6 |
| 39 | High | Absent | Low | 24 | 158.2 | 158.7 | 159.0 | 158.6 |
| 40 | Low | Absent | High | 24 | 257.0 | 259.5 | 258.3 | 258.3 |
| Blank |  |  |  | 24 | 0.0 | 0.0 | 0.0 | 0.0 |
| 1 | High | Present | High | 45 | 133.7 | 132.3 | 130.0 | 132.0 |
| 2 | Low | Absent | High | 45 | 275.6 | 281.1 | 272.6 | 276.4 |
| 3 | Low | Present | High | 45 | 164.2 | 160.9 | 159.8 | 161.6 |
| 4 | High | Present | Low | 45 | 136.8 | 134.7 | 134.9 | 135.5 |
| 5 | High | Absent | High | 45 | 191.7 | 193.2 | 191.7 | 192.2 |
| 6 | Low | Present | High | 45 | 226.9 | 225.0 | 225.9 | 225.9 |
| 7 | Low | Present | Low | 45 | 333.4 | 335.7 | 333.9 | 334.3 |
| 8 | Low | Absent | Low | 45 | 264.7 | 261.3 | 262.5 | 262.8 |
| 9 | Low | Absent | High | 45 | 374.1 | 375.1 | 376.8 | 375.3 |
| 10 | High | Absent | Low | 45 | 318.9 | 309.2 | 307.9 | 312.0 |
| 11 | High | Absent | High | 45 | 281.2 | 282.5 | 282.1 | 281.9 |
| 12 | High | Present | High | 45 | 62.7 | 65.0 | 66.3 | 64.7 |
| 13 | High | Absent | Low | 45 | 158.7 | 158.9 | 159.8 | 159.1 |
| 14 | Low | Present | Low | 45 | 332.2 | 327.8 | 323.6 | 327.9 |
| 15 | Low | Present | High | 45 | 147.0 | 145.5 | 143.3 | 145.3 |
| 16 | Low | Absent | High | 45 | 289.1 | 286.5 | 285.0 | 286.9 |
| 17 | High | Absent | Low | 45 | 313.6 | 315.3 | 312.3 | 313.7 |
| 18 | High | Absent | High | 45 | 244.5 | 242.5 | 245.5 | 244.2 |
| 19 | High | Present | Low | 45 | 84.1 | 83.3 | 85.3 | 84.2 |
| 20 | Low | Absent | Low | 45 | 93.1 | 95.2 | 94.3 | 94.2 |
| 21 | Low | Present | Low | 45 | 131.6 | 130.6 | 122.0 | 128.1 |
| 22 | Low | Absent | Low | 45 | 171.6 | 171.2 | 170.4 | 171.1 |
| 23 | High | Present | Low | 45 | 70.6 | 73.1 | 72.2 | 71.9 |
| 24 | High | Absent | High | 45 | 104.5 | 104.2 | 102.6 | 103.8 |
| 25 | High | Present | High | 45 | 25.9 | 26.8 | 27.2 | 26.6 |
| 26 | High | Absent | Low | 45 | 349.7 | 344.2 | 342.6 | 345.5 |
| 27 | High | Present | Low | 45 | 58.9 | 61.3 | 57.9 | 59.4 |
| 28 | Low | Present | Low | 45 | 107.5 | 107.0 | 107.7 | 107.4 |
| 29 | Low | Present | High | 45 | 133.4 | 132.8 | 131.8 | 132.7 |
| 30 | Low | Absent | Low | 45 | 125.6 | 125.4 | 125.9 | 125.6 |
| 31 | Low | Absent | Low | 45 | 221.7 | 222.2 | 218.5 | 220.8 |
| 32 | Low | Present | High | 45 | 156.1 | 155.4 | 155.4 | 155.6 |
| 33 | Low | Absent | High | 45 | 270.0 | 267.9 | 268.2 | 268.7 |
| 34 | High | Present | High | 45 | 119.6 | 115.5 | 115.9 | 117.0 |
| 35 | Low | Present | Low | 45 | 91.5 | 91.1 | 90.9 | 91.1 |
| 36 | High | Present | Low | 45 | 201.2 | 201.8 | 199.8 | 200.9 |
| 37 | High | Absent | High | 45 | 254.8 | 255.9 | 255.4 | 255.4 |
| 38 | High | Present | High | 45 | 135.2 | 137.7 | 135.5 | 136.1 |
| 39 | High | Absent | Low | 45 | 224.8 | 222.7 | 223.5 | 223.7 |
| 40 | Low | Absent | High | 45 | 212.9 | 215.1 | 214.0 | 214.0 |
| Blank |  |  |  | 45 | 0.0 | 0.0 | 0.0 | 0.0 |
