# Supplementary material for: Road Salts as Environmental Constraints in Urban Pond Food Webs
Source: PLoS One. 2014 Feb 26;9(2):e90168. doi: 10.1371/journal.pone.0090168 (PMC3935972; doi:10.1371/journal.pone.0090168)
Supplement: Table S5 — Tadpole metamorphosis data. (DOC) [file pone.0090168.s005.doc]

| Table S5. Tadpole mean days to metamorphosis, mean mass at metamorphosis (g), fraction of tadpoles reaching metamorphosis and percent survival within the 20 mesocosms receiving tadpoles (n = 30 tadpoles per mesocosm), chloride (high or low) and inoculum source (specific conductance = high or low) treatments. | | | | | | |  | | | |
| --- | --- | --- | --- | --- | --- | --- | --- | --- | --- | --- |
| Pond ID | Chloride | Inoculum | Mean Days to Metamorphosis | Mean Mass at Metamorphosis | Fraction Reaching Metamorphosis | % Survival |  |  |  |  |
| 1 | High | High | 49.23 | 0.3455 | 0.87 | 90 |  |  |  |  |
| 3 | Low | High | 46.40 | 0.3138 | 0.83 | 90 |  |  |  |  |
| 4 | High | Low | 47.14 | 0.3048 | 0.70 | 80 |  |  |  |  |
| 6 | Low | High | 52.25 | 0.3137 | 0.67 | 100 |  |  |  |  |
| 7 | Low | Low | 47.73 | 0.2742 | 0.73 | 73 |  |  |  |  |
| 12 | High | High | 53.27 | 0.3532 | 0.37 | 93 |  |  |  |  |
| 14 | Low | Low | 49.00 | 0.2802 | 0.60 | 90 |  |  |  |  |
| 15 | Low | High | 45.09 | 0.2562 | 0.77 | 97 |  |  |  |  |
| 19 | High | Low | 51.36 | 0.3564 | 0.93 | 100 |  |  |  |  |
| 21 | Low | Low | 46.21 | 0.2790 | 0.97 | 97 |  |  |  |  |
| 23 | High | Low | 55.33 | 0.1967 | 0.20 | 83 |  |  |  |  |
| 25 | High | High | 51.57 | 0.3321 | 0.23 | 53 |  |  |  |  |
| 27 | High | Low | 51.05 | 0.3358 | 0.73 | 93 |  |  |  |  |
| 28 | Low | Low | 50.50 | 0.3166 | 0.73 | 87 |  |  |  |  |
| 29 | Low | High | 47.56 | 0.2242 | 0.60 | 97 |  |  |  |  |
| 32 | Low | High | 51.81 | 0.3333 | 0.70 | 97 |  |  |  |  |
| 34 | High | High | 46.73 | 0.2671 | 0.50 | 87 |  |  |  |  |
| 35 | Low | Low | 55.00 | 0.3658 | 0.30 | 90 |  |  |  |  |
| 36 | High | Low | 45.66 | 0.3651 | 0.97 | 97 |  |  |  |  |
| 38 | High | High | 44.12 | 0.3049 | 0.83 | 93 |  |  |  |  |
